# Pharmacist counseling to cardiac patients in Israel prior to discharge from hospital contribute to increasing patient's medication adherence closing gaps and improving outcomes

**DOI:** 10.1186/1479-5876-10-34

**Published:** 2012-03-01

**Authors:** Bishara Bisharat, Lubna Hafi, Orna Baron-Epel, Zaher Armaly, Abdalla Bowirrat

**Affiliations:** 1 Department of Internal Medicine and Clinical Neuroscience, EMMS Nazareth-The Nazareth Hospital, Nazareth 16100, Israel; 2 School of Public Health Haifa University, Haifa, Israel

**Keywords:** Pharmacist intervention, Cardiac patients, Medication adherence

## Abstract

**Background:**

Medication non adherence is a global epidemic perplexing phenomenon that is eminent, but not insurmountable. Our first objective was to explore whether providing pharmacist's counseling to cardiac patients prior to discharge can increase patient's medication adherence, and our second objective was to assess whether better medication adherence leads to reduction of hospital readmissions.

**Methods:**

Observational study was conducted among diagnosed cardiac patients using an intervention strategy at discharge from two hospitals in Israel; The Nazareth and the Haemek hospital. 74 patients were recruited between January 2010 and January 2011. Two separate groups were selected; intervention group: 33 patients who prior to discharge received nurse, pharmacist interventions, and control group: 41 patients who had received the nurse and hospital discharge counseling only.

**Results:**

Regression analysis for examining the first objective reflected significant effect when having a pharmacist interventions, which explains the increasing 11.6% of the variance in medication adherence, [F change (1,73) = 9.43, *p *< 0.003]. Stepwise regression analysis for examining the second objective demonstrated that the relation between medication adherence and readmissions was insignificant [F _(1,73) _= 9.43, n.s].

**Conclusions:**

While physicians and nurses can have an impact on improving adherence, pharmacists have demonstrated the ability to inform, problem-solve and provide performance support directly to patients.

## Background

### What is new?

Medication adherence among patients in Israel is very low, especially among Cardiac patients.

Pharmacist interventions to cardiac patients prior to discharge from internal medical departments can improve medication adherence by improving their knowledge, and can contribute to improving the effectiveness and benefit from medical treatments, and thus reducing costs of readmissions.

The term adherence is effectively used interchangeably with compliance. They both describe the agreement between the medical regimen prescribed by the treating physician and actual patient practice. However, adherence implies a more active role of the patient in the process and indicates a responsibility of both parties to achieve success, but non-compliance becomes linked with negative connotations [1], so we steer clear of it. Plainly, compliance suggests a process in which dutiful patients passively follow the advice of their physicians. Adherence, in contrast, better fits how most patients actively participate in their care and decide for themselves when and whether to follow their doctor's advice. The failure to adhere to medication instructions either, willful or inadvertent especially among elderly is becoming an international problem, in fact, if medication non-adherence were a disease, it could be termed an "epidemic [2,3].

Indeed, it is a puzzling phenomenon, with eminent dreadful consequences onto health system, but insurmountable merely by using calculated strategies. With each passing day, tremendous intentions is being made to understand the core reasons for non-adherence and design programs that will tackle these issues [4].

Also, there has been a realization by all concerned stakeholders that they have to stop viewing non-adherence as either 'my problem' or 'their problem' and treat it as 'our problem'.

Medication non-adherence is a serious problem facing the health system in general and the medical staff specifically. It is among the leading causes for hospital readmissions, and it was documented that non-adherence is responsible for more than 50% of the readmissions among cardiac patients [5].

Among chronic illnesses, especially heart diseases, costs are largely derived from expensive exacerbations that require emergency visits and hospitalizations [6].

It has been shown that 27% of patients hospitalized for Heart Failure (HF) were re-hospitalized within 90 days, with most of them as a result of medication or dietary non-adherence [7].

Nevertheless, adherence is an umbrella term used to embrace various components involved in the process of patients taking medication as prescribed [8].

Among the documented reasons for medication non-adherence are lack of education and forgetfulness [9]. Patients' report of non-adherence includes that several changes are made to their usual treatment regimen and they are suddenly forced to take responsibility for their own health [10]. Moreover, less than 20-25% of the respondents reported knowledge about side effects and required lifestyle changes. Additional risk factors to such behavior are: the use of large number of prescribed medications, the use of a large number of daily doses [11].

Indeed, complexity of medication regimens may adversely affect adherence and treatment outcome. The results of several studies have shown that, as medication regimen complexity increases, adherence declines. Medication complexity includes items on frequency, special instructions and behaviors related to medications, and other difficulties encountered [12-15].

The degree of adherence varies across diseases, but for many chronic diseases, 40% to 50% of patients do not persist with initial treatments beyond 12 months [16-18].

In hypertension and cardiovascular risk management adherence, rates at one year average about 60%, undoubtedly contributing to the less than optimal blood pressure control and less than optimal clinical outcomes of these patients [19]. In fact, adherence to medical recommendations is a multifactorial behavior and requires a multimodel response [20]. Therefore, strategies to encourage adherence must not only address personal and cognitive factors such as knowledge of the regimen, belief in benefits of treatment, subjective norms, and attitudes toward medication-taking behavior, but also environmental and social factors such as the interpersonal relationship between the provider and the patient [20].

As we search for strategies and solutions geared toward improving patient adherence to prescription drug therapy, it is imperative to involve pharmacists. While physicians, nurses and other healthcare professionals can moderately have an impact on improving adherence alone, the collaboration of pharmacists, as the medication experts have demonstrated elevated ability to inform, problem-solve and provide performance support directly to and with patients. Since, medication dispensing is the best-known function of the pharmacist, pharmacists--through counseling, from filling the initial script to completion, medication therapy management, disease-state management, and other means--can play a crucial role in patient care.

There are opportunities in every type of pharmacy practice to improve patients' adherence and therapeutic outcomes, and pharmacists must embrace and act according to them [21].

In this study we are looking to examine the role of the pharmacists as healthcare professionals who can improve adherence, since a few studies have been conducted on evaluating the contribution of pharmacist medication counseling to patients when discharged from hospitals. Pharmacists can play a quintessential role in initiating counseling to patients and in monitoring their adherence in order to improve their treatment outcomes. Education and counseling regarding medication therapy given to patients at discharge has been shown to affect demand and utilization of health services along several dimensions.

Globally, pharmacist-based patient education is growing, in part because pharmacists are appreciated to be "in a privileged position, with their expertise in pharmacological treatment, to provide education, identify medication adherence issues and counsel the patient" [22]. In addition, many pharmacists utilize shared databases and information system capabilities that facilitate the flow of information and generate improved clinical outcomes and cost benefits in the management of diseases like hypertension [23,24], diabetes and cardiovascular disease [25,26]. In one unique Canadian trial to improve treatment adherence among patients with hypertension, hypercholesterolemia and heart failure, 824 patients were enrolled by their pharmacist in two Ontario communities [27].

The study includes in the control setting (n = 28 pharmacies), and in the intervention setting (n = 26 pharmacies). Patients were followed at 3 month intervals, for an average duration of 269 days. For lipid lowering therapy, the intervention was associated with an increase of 13% in patient adherence (*p *< 0.005) and for hypertensive patients, the impact was an additional 8% adherence. Interestingly, in both control and intervention sites, patients had higher than usually reported adherence rates [27].

The purpose of our study is to shed light on the underlying causes and consequences of non-adherence in general, highlighting the impact of different variables, such as the demographic characteristics, ethnicity, sex, age and socioeconomic status, complexity of treatment, patient self-efficacy, social support, disease knowledge, treatment alternatives, costs and side effects and disruption of patients' life style, and specifically to explore the strategy whether providing pharmacist counseling to cardiac patients in Israel, prior to discharge from internal departments, can contribute to increasing patient's medication compliance. Since, cardiac patient's non-adherence documented high rate of readmissions exceeds roughly 50% countrywide and their coping skills to face often-fragile condition aggravate their sufferance substantially and they are particularly at-risk from drug non-adherence and potentially jeopardize their health and longevity. Two essential target goals were taken in mind for this research: The first objective was to explore whether providing pharmacist interventions and supports for patients with heart failure prior to discharge leads to better medication compliance in comparison to patients discharged with no such interventions?, and the second goal was if we have better medication compliance; this aim leads to less readmissions?.

## Methods

This observational study was conducted among diagnosed Chronic Heart Failure (CHF) patients using a pharmacists intervention strategy which was operated at the time of discharge from internal medical department, from two Medical Centres in Israel; EMMS Nazareth-The Nazareth Hospital, and in Haemek Medical Center in Afula, after its approval by the ethical committees of both hospitals and after obtaining signed written informed consent at the study enrollment from all participants.

A sample of 74 patients were recruited for the study, which formed two separate groups: an *intervention group*, where patients, prior to discharge, received nurse counseling and afterwards pharmacist interventions, which took an average time of 20-30 minutes and a *control group*, who had received the routine discharge program that was provided by the hospital and discharge counseling only by a nurse. These patients were taken for data collection over a period of one year (from Jan. 2010 to Jan. 2011). There was no restriction of sex, marital status, educational level, ethnic group, socioeconomic status and place of residence. Exclusion criteria was illiterate patients, severely hearing impaired or demented, patients not prescribed chronic medications before hospitalization, patients with a coexisting terminal illness such as cancer or chronic renal failure requiring dialysis, patients who were to be discharged to another health-care facility, patients who were not able to take their medication independently, and who didn't have assistance from a relative or a home care giver and pregnant women. Patients who were discharged from Nazareth Hospital and from Haemek Medical Center and who followed the inclusion criteria were enrolled into the intervention group. The control group was determined using a history list of previously discharged patients from these two medical centers and who followed the inclusion criteria. All information regarding age, sex, number of chronic medications they take were obtained using Clalits' health services "Ofek" computer program.

Basic demographic information along with complete medical history, type of treatment and the different reasons for non-adherence were recorded. A senior physician interviewed the patients using a questionnaire that was designed for this purpose. The pharmacist emphasized the importance of adherence and what to do if a dose was missed. She/he also emphasized the importance of contacting the physician whenever feeling any unusual change in overall health. By the end of the counseling the pharmacist asked the patient about discrepancies between her/his and the nurse's counseling. When there were any, the nurse was called and reconciliation was made at the moment. The pharmacist used the Thompson Micromedex^® ^healthcare series for getting all the necessary and desired information regarding her/his counseling. The correlation between medication complexity and the parameters of age, gender, underlying disorder and the number of medications were determined using the Medication regimen complexity index (MRCI), which comprises three sections:

(1) Nature of dosing forms, that is, whether it is a tablet/inhaler/gel.

(2) Dosing frequency, that is, how often the medication is taken.

(3) Additional directions that need to be followed, that is, take before or after a meal/at a specific time/breaking the tablet in half.

All entries on the MRCI were based on information from our 74 participants' scripts reviewed. If a script contained more than one medication, the MRCI was completed for each different medication. The MRCI is an open-ended index, thus there would be no limit to the total number of medications that could be included on the MRCI. Data was analyzed using SPSS (Statistical program for Social Sciences version 15.1). Chi-square tests were used to compare gender, age, underlying conditions and the number of medications.

### Data collection

Each month, the research pharmacist looked for data regarding each patient's prescriptions filling by using the information registered in a Clalits' health services "Ofek" computer program. The researcher checked the following:

1. Whether the patient filled the prescription each month regularly. Dates of prescription fillings were registered.

2. When there were changes in two medications or more the participant was followed tightly and extensive clarification and explanation was provided to him/her to insure full understanding of the medical regime changes to avoid confusions. When there was a replacement of a drug with another of the same therapeutic group, the follow up was made to the new drug.

3. The researcher measured the intervals between refills and compared the value to the days' supply of medication dispensed.

Furthermore, readmissions for both group patients were monitored for the period of 6 months, and hospitalization costs were calculated using a list of hospitalization costs in internal departments and intensive care units. The dependent variables measured were: The number of chronic medications, medication compliance and the number of readmissions. Other dependent variables such as complexity of medication regimen, socioeconomic situation, including income and education level were evaluated.

### Adherence assessment

Monthly, each patient's prescription filling was monitored through a Clalit "Ofek" computerized program which recorded all health information regarding patients. It also included pharmacy prescription fillings. The sum of the days' supply obtained over a series of 6 months was calculated for each medication and was divided by the total days from the beginning to end of the time period (Table 1). This ratio is named *Medication Possession Ratio (MPR)*.

**Table 1:** Gives the example if a patient for a period of 3 months has taken the following days' supply

Prescription interval	Day of fill	Days' supply obtained	Days in interval
1	0	30	30

2	30	30	30

3	60	30	90

Then his adherence would be:

$$\begin{array}{c} \frac{\left(30+30+30\right)\left(\text{Total}\;{\text{days}}^{\prime }\;\text{supply}\;\text{obtained}\right)}{\left(30+30+90\right)\left(\text{Total}\;{\text{days}}^{\prime }\;\text{in}\;3\;\text{intervals}\right)}=0.6^{*}100=60\% \end{array}$$

An MPR of 80% is a reasonable threshold for adherence because it suggests very few days without drug on hand and, consequently, fairly continuous medication usage [20]. Values greater than 80% were categorized as good adherence and values less than it, were categorized as non-adherence. A mean adherence for each patient and for each group was calculated for further analysis.

### Readmissions assessment

Each patient was monitored for hospital readmissions; we reviewed the medical record for each patient in order to confirm the admission date and reason. Each patient's total days of hospitalization were documented. Total number of readmissions was calculated. A comparison was made between the two groups to assess the differences.

### Study variables

The dependent variables were:

1. Medication adherence, which is dependent on the pharmacist counseling, and on other variables such as age, sex and complexity of medication regimen. Other variables that may affect the medication adherence which was not taken into consideration due to difficulty obtaining such information by using the "Ofek" computer program are status of education, and socioeconomic status.

The variable was calculated according to the equation that was mentioned in the literature review.

2. Number of readmissions, which is dependent on medication adherence.

The variable was obtained by using Clalits' health services "Ofek" computer program: each month we looked for registered readmissions in different hospitals in Israel.

The independent variables were:

1. Complexity of medication regimen.

2. Socioeconomic situation, including income

3. Education level

4. Pharmacist counseling

5. Number of chronic medications

6. Age

7. Sex

### Possible bias

a. Lost to follow up patients.

This bias can be dealt with by using the intention to treat analysis.

### Statistical analysis

For the statistical analysis the SPSS 14.0 software was used, the statistical significance was set at bilateral 5%, in addition, patient characteristics and responses were summarized with descriptive statistics including frequency, mean, SD, and range. Responses on the measure of medication adherence were dichotomized as "compliant" and "non-compliant." A stepwise regression was conducted in order to check the first hypothesis: as the 1^st ^step, the independent variable of pharmacist counseling was inserted into regression, followed by the other variables: medication regimen complexity, age and sex variables. A stepwise regression was conducted in order to check the second hypothesis: as the 1st step the dependent variable of medication adherence was inserted, followed by the complexity of the medication regimen, age and sex variables. Crosstabs were built and relations between medication adherence variable and the age, sex, number of readmissions and complexity of medication regimen were obtained. Complexity of the medication regimen for outpatients was assessed using the Medication regimen complexity index (MRCI), a tool used to assess medication complexity developed by George et al. [28].

## Results

In this descriptive study performed in the two medical centers 74 patients were included (33 intervention group and 41 control group). Mean age of intervention group was (65.3 ± 12.17), were males represent 22 (67%) and females form 11(33%). The number of chronic medication was [(7 ± 2.7), (range: 3-17)], number of readmissions [(0.7 ± 1.18), (range: 0-5)]. The adherence percentage was [(77.83 ± 22.85), (range: 22.22-105.1)].

The control characteristics were as follow: mean age ± SD [(72.73 ± 10.64), range (36-90)], were males represent 28 (68%) and females 13(32%). The number of chronic medication was [(6.59 ± 1.9), (range: 3-11)]. The number of readmissions was [(0.93 ± 1.01), (range: 0-3)]. The adherence percentage was [(62.46 ± 24.5), (range: 6.25-100)], (Table 2).

**Table 2:** Characteristics of the Intervention group (n = 33) and of the Control group (n = 41)

Characteristics	Intervention group values	Control group values
Age		
Mean ± SD	65.3 ± 12.17	72.73 ± 10.64
Range	41.92	36-90

Sex		
Male	22 (66.7%)	28 (68.3%)
Females	11 (33.3)	13 (31.7%)

Number of chronic medications		
Mean ± SD	7 ± 2.71	6.59 ± 1.92
Range	3-17	3-11

Number of readmissions		
Mean ± SD	0.7 ± 1.18	0.93 ± 1.01
Range	0-5	0-3

Adherence percentage		
Mean ± SD	77.83 ± 22.85	62.46 ± 24.52
Range	22.22-105.10	6.25-100

By dividing patients according to gender, number of chronic medications, medication adherence, number of readmissions as shown in (Table 3), the distribution of variables among males (n = 50), were as follow: number of chronic medications was (6.66 ± 2.43), medication adherence was (69.20 ± 26.83) and number of readmissions was (0.88 ± 1.02). The distribution of variables among females (n = 24) was as follow: Number of chronic medications was (7 ± 2.02), medication adherence was (69.54 ± 20.66) and number of readmissions was (0.71 ± 1.23).

**Table 3:** Shows the distribution of variables in both gender

	Age	Number of chronic medications	Medication adherence	Number of readmission
Males N = 50				
Mean ± SD	76.9 ± 12.27	6.66 ± 2.43	69.20 ± 26.83	0.88 ± 1.02
Minimum	36	3	6.25	0
Maximum	90	17	100	3

Females N = 24				
Mean ± SD	72.6 ± 10.49	7 ± 2.02	69.54 ± 20.66	0.71 ± 1.23
Minimum	52	4	24.98	0
Maximum	92	11	105.1	5

1 For the statistical analysis (Chi-squared test) using SPSS 14.0 software was performed

2 The statistical significance level that was used was bilateral 5%

Comparing the variables between both genders there were no statistical significant differences. Table 4 summarizes the distribution of age, number of chronic medications, medication adherence, and number of readmissions, in both the intervention and control groups, the results was also statistically insignificant.

**Table 4:** Summarizes the distribution of variables in both the intervention and control groups

	Age	Number of chronic medications	Medication adherence	Number of readmission
Intervention group N = 33				
Mean ± SD	65.33 ± 12.12	7 ± 2.71	77.83 ± 22.85	0.7 ± 1.18
Minimum	41	3	22.22	0
Maximum	92	17	105.10	5

Control group N = 41				
Mean ± SD	72.73 ± 10.64	6.59 ± 1.92	62.45 ± 24.52	0.93 ± 1.01
Minimum	36	3	6.25	0
Maximum	90	11	100	3

1 For the statistical analysis (Chi-squared test) using SPSS 14.0 software was performed

2 The statistical significance level that was used was bilateral 5%

Conducting regression analysis for examining the first hypothesis: "We assumed that patients with Heart Failure who had pharmacist counseling will have better medication adherence than patients who had merely nurse counseling," while taking into consideration the effect of the differences in the complexity of the medication regimen (e.g. number of chronic medications each patient have), their age and their sex. We found after analyzing the regression, that having a pharmacist counseling in the intervention group explains 11.6% of the variance in medication adherence, [F change (1,73) = 9.43, *p *< 0.003], in comparison with the control group. We can also notice that this variance that is explained by the medication adherence is correlated to the pharmacist counseling while the complexity of the medication regimen, the age of the patient and the sex don't contribute to the ability of predicting this variance (Tables 5 and 6). Examining the second hypothesis (if we have better medication adherence; this hypothesis leads to less readmissions), while also taking into consideration the effect of the differences in medication regimen complexity, age and sex. Table 7 represents the results of stepwise regression after inserting the four independent variables which they predict readmissions. By analyzing the stepwise regression, it is noticed that the relation between medication adherence and readmissions is not statistically significant [F _(1,73) _= 9.43, n.s]. None of the other variables can explain the variance in the number of readmissions (Table 6).

**Table 5:** Describes the results of a stepwise regression after inserting of all variables

Variables	r
Pharmacist Intervention	0.34***

Number of chronic medications	0.034 -

Age	0.052 -

Sex	0.062 -

**Table 6:** Compares the variance in medication adherence after having a pharmacist in the intervention group versus the control group

Step	Predicting variable	R^2^ Change	R2	F Change	F	t	β
1	Pharmacist counseling	0.116	0.116	9.43 ***	9.43***	3.071***	0.37 ***

*** *p *< 0.05 n = 74

**Table 7:** Represents the results of stepwise regression analysis after inserting the independent variables

Variables	r
Pharmacist counseling	0.141 -

Number of chronic medications	0.131

Age	0.054

Sex	0.074 -

By analyzing the stepwise regression, it is noticed that the relation between medication adherence and readmissions is not statistically significant [F _(1,73) _= 9.43, n.s]. None of the other variables can explain the variance in the number of readmissions

## Conclusions

Plethora of studies has confirmed that the rate of non-adherence is higher in patients with chronic illnesses worldwide [29,30]. It is universally accepted that 50% to 75% of patients are non-adherent [31], which undermines their care and leads to increased health care costs [32], morbidity, and mortality [33]. This is partially because the drug regimens for these patients are often long-term, complex thus altering existing behavioral patterns. The World Health Organization has estimated that, even in affluent countries with well-developed health care infrastructure, adherence to long-term medication therapy for chronic illness averages only 50% [34].

We have conducted a study whose aims were directed toward increasing patients' medication adherence and as a result, decreasing their number of readmissions through pharmacist-directed patient education (Table 2). This study focused specifically on patients with heart failure who, according to the literature, are at higher risk for early readmissions [35].

In our study we aimed to examine the role of the pharmacists as healthcare professionals who can improve adherence enhancement, since a few studies have been conducted on checking the contribution of pharmacist medication counseling to patients when discharged from hospitals. We found that pharmacists can play a quintessential role in initiating such an education to patients and in monitoring their adherence in order to improve their treatment outcomes. Education and counseling regarding medication therapy given to patients at discharge has been shown to affect demand and utilization of health services along several dimensions.

Indeed, we support the idea that incorporating a pharmacist as part of the health providing team, especially in hospitals, is crucial and contribute to both improving patients' health and reducing the burden over the health system. Moreover, our pharmacist interventions explained 11.6% of the variance in patients' medication compliance in the intervention group in comparison to the control group (*p *= 0.03).

Our research is in accordance with other opinions of the indisputable fact that non-adherence is a serious medical issue that may lead to death and elevated costs, both for patients and providers and it is hard to identify just one intervention for non-adherence because non-adherence is different in all patients. Thus, no single strategy appears to be the best, due to the multitude of factors that affect a patient's decision to adhere to the prescribed medications. In fact, adherence with medication is a challenge that faces the whole population whether they are patients, medical professionals or both.

Concomitantly, we never lost hope to look for an alternative strategies that might enhance the adherence and decreases the readmissions through pharmacist-directed patient education. We assume the motivation is something that we can control. However, motivation is internal. All we can do is educate an individual and support them to be motivated. In fact, remove barriers to adherence, and increasing confidence, communication between doctor and patient and trying to explain the regimen complexity may help to improve the outcome [36,37]. Furthermore, researchers suggest that pharmacists, having direct contact with patients while patients are engaged in their medical regimen, have a better ability to detect adherence problems. A new stream that is being proposed to improve patient adherence is to implement a system around the community-based pharmacist. A community-based pharmacist is one who has direct involvement in a patient's treatment plan, has direct and frequent contact with physicians, and has an active role in changing or altering a patient's medical regimen [38].

After studying the literature, one can only conclude that there is still no real consensus concerning the most effective way to improve patient adherence. The research shows that adherence to medications are not routinely measured in clinical practice and that a universal standard that can be easily implemented has not been established [39-49]. Our study results are in harmony with previous studies in emphasis the role of the pharmacists in CHD patients' management [27]. This synchronization spring up by the pharmacist counseling contribution via increasing the patient education and knowledge concerning their medication adherence.

### Study limitations

1. The Medication Possession Ration (MPR) method for calculating each patient's adherence may not be completely conveying the true situation. Early refilling would lead to an MPR of more than 100%. In such case, the MPR is often truncated at the maximum value of 100% indicating the potential for perfect adherence. Moreover, for some medication, the MPR value might be more than 100%, reflecting inappropriate medication adherence, and when calculating a mean MPR, this might shift the whole value towards high medication adherence when in fact the patient was not adherent in other medications.

Moreover, although the MPR provides an insight into the availability of medications, it doesn't provide information on the timeliness and consistency of refilling. This is true only if the gaps between refills are known to be small and insignificant.

2. It is assumed that a new refill of a prescription implies complete ingestion of the previous refill. However, the possibility exists that an individual may be providing mediations to others, dumping medications before refilling, or stockpiling medications for future usage.

3 The selection of a cut-off value directly affects the measurement's accuracy of providing information on the continuity of medication usage.

4. The sample size may be too small in order to obtain more statistically significant results.

### Strengths of the study

Medication adherence among patients in Israel is very low, especially among Cardiac patients. Defining a practical education method will help increase the knowledge of each patient regarding the medication he or she takes, and thus improves their medication adherence. This will help patients to benefit from their medical regimen which will lead to a decrease in the rate of readmissions.

Pharmacist counseling of Cardiac patients prior to discharge from internal departments can improve medication adherence by improving their knowledge, and can contribute to improving the effectiveness and benefit from medical treatments, and thus reducing costs accompanied to readmissions.

In summary, medication adherence is an important persistent problem and certain interventions could be effective in improving medication use after discharge. Pharmacists are the critical piece for elevating the discussion of medication adherence--with both other medical professionals and directly with patients. These highly trained professionals are among the nation's most respected professional and among the most accessible to consumers. Solutions should not only help design scientifically-based, proven intervention strategies, but also reduce the time and cost involved with implementing these strategies in various healthcare settings.

Behavior change requires different strategies for different people. Trust, hope, fear, motivation, knowledge, literacy, skills, tools, rehearsal, reinforcement, feedback, confidence and competence are key concepts in the literature of medication adherence. The problem of non-adherence often defies isolated efforts and tactics, but studies have shown that multi-disciplinary approaches work well, multiple channels work better than single channels, and a longer time horizon is more realistic than a short one. Indeed everyone is different, and everyone changes over time.

## Competing interests

The authors declare that they have no competing interests.

## Authors' contributions

BB - carried out the study design and participating in the recruitment of patients in addition participated in editing the manuscript and finding the manuscript. LH - Participated in the study design, and performed the tables and figures and helped in performing the statistical analysis. OB - participated in the collections of data and study design, editing the manuscript and participated in writing part of the introduction. ZH - Participated in writing part of the conclusion and study design, editing the manuscript, and in helped to draft the manuscript. BA - Conceived the study, and participated in its design and coordination and wrote almost all manuscript (introduction & discussion) and helped to draft the manuscript. All coauthors have read and approved the final manuscript.
